# Temporal dynamics of selective attention and conflict resolution during cross-dimensional go-nogo decisions

**DOI:** 10.1186/1471-2202-8-68

**Published:** 2007-08-17

**Authors:** Bruno Kopp, Sandra Tabeling, Carsten Moschner, Karl Wessel

**Affiliations:** 1 Research Institute of Cognitive Neurology, Klinikum Braunschweig, and University of Technology Carolo-Wilhelmina at Braunschweig, Salzdahlumer Str. 90, 38126 Braunschweig, Germany

## Abstract

**Background:**

Decision-making is a fundamental capacity which is crucial to many higher-order psychological functions. We recorded event-related potentials (ERPs) during a visual target-identification task that required go-nogo choices. Targets were identified on the basis of cross-dimensional conjunctions of particular colors and forms. Color discriminability was manipulated in three conditions to determine the effects of color distinctiveness on component processes of decision-making.

**Results:**

Target identification was accompanied by the emergence of prefrontal P2a and P3b. Selection negativity (SN) revealed that target-compatible features captured attention more than target-incompatible features, suggesting that intra-dimensional attentional capture was goal-contingent. No changes of cross-dimensional selection priorities were measurable when color discriminability was altered. Peak latencies of the color-related SN provided a chronometric measure of the duration of attention-related neural processing. ERPs recorded over the frontocentral scalp (N2c, P3a) revealed that color-overlap distractors, more than form-overlap distractors, required additional late selection. The need for additional response selection induced by color-overlap distractors was severely reduced when color discriminability decreased.

**Conclusion:**

We propose a simple model of cross-dimensional perceptual decision-making. The temporal synchrony of separate color-related and form-related choices determines whether or not distractor processing includes post-perceptual stages. ERP measures contribute to a comprehensive explanation of the temporal dynamics of component processes of perceptual decision-making.

## Background

We are only beginning to understand the neural mechanisms of decision-making although decision-making plays a pivotal role in translating perception into action [1]. Adaptive performance requires decision processes capable of identifying perceptual situations demanding specific responses. Performance emerges from interaction between an organism's goals (top-down influences) and stimuli that impact on that organism (bottom-up influences). Decision processes therefore must be able to resolve possible conflicts between stimulus-driven (reflexive) and goal-contingent (voluntary) control over performance. This study aims at describing the temporal dynamics of neural processes related to component processes of perceptual decision-making (perceptual analysis, selective attention, conflict-related processing) in humans.

The issue of stimulus-driven versus goal-contingent control has traditionally been important for theoretical formulations of selective attention [2-4]. Reflexive and voluntary factors interact to allow for selectivity of visual attention [5]. Several authors argued that visual selective attention is predominantly stimulus-driven [6-8]. This view proposes that salient visual events attract attention irrespective of the goals of the observer. However, observers often adopt a deliberate voluntary attentional set for target-compatible features when they are searching for particular visual target stimuli [9]. Purely goal-contingent capture of visual attention has been demonstrated when to-be-ignored non-target stimuli (distractors) possess target-defining features [10]. Theories of attention have generally been divided between early-selection theories, which propose that attention influences perceptual processes, and late-selection theories, which propose that attention operates only after perception is complete [11].

This study was specifically designed to investigate the dynamic coordination of neural processes related to perceptual decision-making. Visual feature analysis, selective attention and conflict-related processing were analyzed by event-related brain potentials (ERPs). ERPs provide online measures of cortical processing of events with excellent temporal resolution, even when the events do not require behavioral responses [12]. The best-described ERP index of attention is the *P3b *component which is a parietal positivity, peaking at approximately 400 ms after stimulus onset. It is larger to infrequent stimuli, particularly when these stimuli are targets. The P3b can be elicited by stimuli that differ in their probability of occurrence, but also in the amount of goal-relevant information presented by the stimulus [e.g., [13,14]].

There are two additional families of earlier ERP components which also show goal-contingent enhancement to target stimuli. Firstly, feature-based attention as assessed by ERPs affects processing in visual cortical areas [15]. This modulation, termed *selection negativity (SN)*, was found for a variety of visual features, including color, shape, and spatial frequency. The SN is observed over the parieto-occipital scalp, beginning at around 150 ms and lasting 200 ms or more [16]. The cortical sources of the SN are probably located in the corresponding feature-selective areas of the extrastriate cortex [17]. Secondly, prefrontal positivities at about the same latency were described, variously termed *P2a *[18], P3f [19], or frontal selection positivity (SP) [20], which is enhanced to visual target stimuli [21]. There is little evidence regarding which cognitive process the P2a reflects [e.g., [22]]. Potts [21] proposed that the P2a reflects stimulus evaluation in the service of the task demands, i.e., an early, perhaps preliminary, target identification mechanism.

Distinctive distractor-contingent components have also been described in various studies. Firstly, infrequent stimuli that are irrelevant to the task (distractors), but that are more salient than the targets, evoke the 'novelty P3' or *P3a *[23,24], which peaks earlier than the P3b and has a more frontocentral scalp distribution than the P3b. The P3a was conceived as a correlate of attentional orienting to salient distractors [25]. This idea finally led to the attention switching model of the P3a which proposes that this ERP component reflects involuntary switching of attention to deviant events [26]. The attention switching model specifically holds that the task irrelevance of the deviant stimulus is an antecedent condition for eliciting the P3a. Debener et al. [27] showed that the task irrelevance of the deviant stimulus is not an antecedent condition for eliciting the P3a. We have shown that the P3a is a neural correlate of shifts between attentional sets in multidimensional visual selection tasks [28]. Secondly, stimulus displays containing response-incompatible distractors, as in flanker, Stroop or certain no-nogo tasks, elicit the *N2c *which has a midline frontal scalp distribution, and peaks around 250–300 ms post-stimulus in tasks that utilize simple stimuli [29-31]. It may be generated in the medial prefrontal cortex [e.g., [32]]. The N2c amplitude is related to the degree of response conflict in correct-response trials, even when no overt signs of response conflict are present. We and others have suggested that the N2c may reflect the detection and suppression of to-be-inhibited responses [29,33].

**Figure 1:**
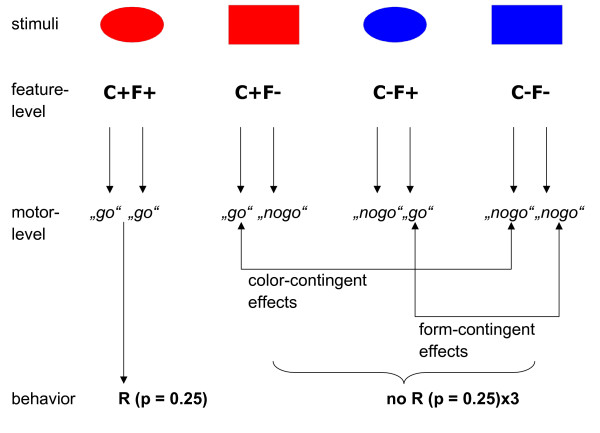
**Task design**. The task uses four different types of stimuli (uppermost panel), each of which is composed of two features (a color, C, and a form, F). Three levels of processing are envisaged, i.e. the feature-level, the motor-level, and behavior. The target, C+F+, demands a response, whereas all non-targets require not to respond. When the target object is the red ellipse, C+F+, the red rectangle is the color-overlap distractor, C+F- (composed of the target-compatible color feature, C+, and the target-incompatible form feature, F-), the blue ellipse is the form-overlap distractor, C-F+ (composed of the target-incompatible color feature, C-, and the target-compatible form feature, F+), and the blue rectangle serves as standard distractor, C-F- (composed of the target-incompatible color feature, C-, and the target-incompatible form feature, F-). Attentional modulation by target color (C+) can be analyzed by comparing the ERP over the parieto-occipital scalp in response to the color-overlap distractor and the standard distractor, i.e. ERP_C+F- _vs. ERP_C-F-_. Attentional modulation by target form (F+) can be analyzed by comparing the ERP over the parieto-occipital scalp in response to the form-overlap distractor and the standard distractor, i.e. ERP_C-F+ _vs. ERP_C-F-_. R = response.

We examined cortical activity (as measured by ERPs) during go-nogo choices in humans. Prefrontal P2a, parietal P3b, posterior SN, prefrontal N2c and frontocentral P3a were analyzed in the context of a newly developed cross-dimensional perceptual decision-making task. Perceptual decisions that are based on the conjunction of cross-dimensional stimulus features, such as color and form, have been examined in several studies before [e.g., [34,35]]. Figure 1 illustrates our cross-dimensional decision-making task. The task made use of a set of four stimuli. This set was produced by factorially combining two features of two dimensions (color and form). Each participant was instructed that one stimulus was the target (denoted C+F+, i.e., the stimulus with the target-compatible color and with the target-compatible form). It represented the object that demanded a behavioral response. There were three different types of non-target stimuli, a color-overlap distractor (C+F-, i.e., the stimulus with the target-compatible color and with the target-incompatible form), a form-overlap distractor (C-F+, i.e., the stimulus with the target-incompatible color and with the target-compatible form), and a standard non-target stimulus (C-F-, i.e., the stimulus with the target-incompatible color and with the target-incompatible form). In the example of Figure 1, the red ellipse served as the target stimulus, the red rectangle was the color-overlap distractor, the blue ellipse was the form-overlap distractor, and the blue rectangle equaled the standard non-target stimulus.

Figure 1 also depicts the logic of the application of ERPs to this cross-dimensional visual target-identification task. Three levels of processing are envisaged, including the feature-level (uppermost panel), the motor-level and behavior (lowest panel). Attentional modulation by target color (C+) can be isolated by comparing ERPs over the parieto-occipital scalp in response to the color-overlap distractor and the standard distractor, i.e. ERP_C+F- _vs. ERP_C-F-_. Attentional modulation by target form (F+) can be isolated by comparing ERPs over the parieto-occipital scalp in response to the form-overlap distractor and the standard distractor, i.e. ERP_C-F+ _vs. ERP_C-F-_. The SN can be applied to discern color-based and form-based attentional modulation of visual feature analysis. Exactly the same comparisons can be employed to reveal neurophysiological indices of conflict-related processing. Both feature-overlap distractors, but not the standard non-target, possess the potential to provoke response conflict, due to the coexistence of "go" and "nogo" signals (Figure 1). The neural mechanisms of conflict-related processing can thus be analyzed by comparing ERPs (N2c, P3a) over the frontocentral scalp in response to color-overlap and form-overlap distractors, respectively, in comparison to the standard distractor.

The ability to change over time is the very essence of attention [36]. We manipulated the discriminability between stimulus colors at three levels in order to challenge the adaptive dynamics of selective attention. Perceptual decisions should be primarily based on object color if color is a preeminent feature of the to-be-identified targets. The form feature should gain importance when the discriminability between target and distractor colors decreases. We recorded ERPs to examine whether or not color-based and form-based effects, in particular on SN, N2c and P3a, are in accordance with the predicted selection priorities.

## Results

### Response speed and accuracy

Accuracy and response speed are documented in Table 1. Participants performed the required classification at a near-perfect level, as revealed by the hit rates (all mean percentages ≥ 99.5) as well as by the correct rejection rates (all mean percentages ≥ 99.4). A one-way distinctiveness ANOVA was performed on the hit rates (*F *(2, 46) = 1.4, *MSE *= 0.006, *η*^2 ^= 0.06, *p *= 0.267, *ε *= 0.97) and on the average correct rejection rates (*F *(2, 46) = 0.1, *MSE *= 0.002, *η*^2 ^= 0.01, *p *= 0.856, *ε *= 0.94). Accuracy was generally near-perfect, without any modulation by the perceptual distinctiveness of the color dimension. Response times were prolonged in the perceptual hard condition compared to the other perceptual distinctiveness conditions. The readily identifiable slowdown was confirmed by a one-way distinctiveness ANOVA on the response times (*F *(2, 46) = 8.5, *MSE *= 38458, *η*^2 ^= 0.27, *p *< 0.01, *ε *= 0.79). Simple contrasts revealed that response speed was slower in the perceptual hard condition than in each of the remaining perceptual distinctiveness conditions (perceptual hard vs. easy condition: *F *(1, 23) = 8.5, *p *< 0.01; perceptual hard vs. intermediate condition: *F *(1, 23) = 11.1, *p *< 0.01).

**Table 1:** Response speed and accuracy

	easy		intermediate		hard	
			
	M	SD	M	SD	M	SD
RT	390	47	387	52	406	48
hits (C+F+)	99.5	.8	99.5	1.2	99.8	.5
CR (C+ F-)	100	0	99.8	.5	99.7	.6
CR (C-F+)	99.4	.7	99.4	.8	99.6	.7
CR (C-F-)	99.9	.3	99.9	.2	99.9	.3
CR (avg)	99.7	.3	99.7	.3	99.7	.3

RT, response time (in ms); hits, (as a percentage); CR, correct rejections (as a percentage); C+F+, target stimulus; C+F-, color-overlap distractor; C-F+, form-overlap distractor; C-F-, standard non-target stimulus; avg, average across C+ F-, C-F+, and C-F-, i.e., across all non-target stimuli.

### P2a and P3b

**Figure 2:**
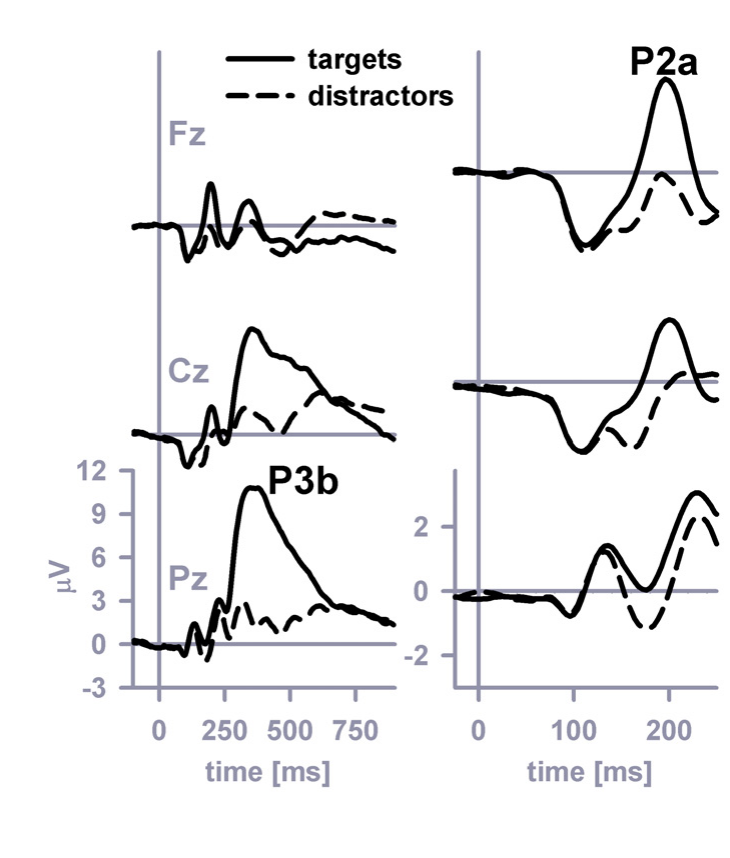
**P3b and P2a at midline electrodes**. The grand-average P3b (left panel) and grand-average P2a (right panel) evoked by targets and non-targets at midline electrodes.

Figure 2 plots grand-average ERPs at midline electrodes that were obtained in response to target stimuli compared to ERPs obtained in response to the average of all non-target stimuli. The target stimuli (+10.8 μV mean amplitude at Pz in the latency range 372–380 ms), but not the non-target stimuli (+1.2 μV mean amplitude at Pz in the latency range 372–380 ms), evoked a readily identifiable P3b (Figure 2, left panel) with parietal maximum. The presence of a target-P3b at Pz was confirmed by a two-way stimulus category (target, non-target) × distinctiveness ANOVA (stimulus category effect, (*F *(1, 23) = 146.3, *MSE *= 22.53, *η*^2 ^= 0.86, *p *< 0.001), with no effect of perceptual distinctiveness or of the interaction between stimulus category and distinctiveness.

The target stimuli (+2.9 μV mean amplitude at Fz in the latency range 188–196 ms), but not the non-target stimuli (-0.1 μV mean amplitude at Fz in the latency range 188–196 ms), evoked a readily identifiable P2a (Figure 2, right panel) with prefrontal maximum. The presence of a target-P2a at Fz was confirmed by a two-way stimulus category (target, non-target) × distinctiveness ANOVA (stimulus category effect, (*F *(1, 23) = 34.9, *MSE *= 9.32, *η*^2 ^= 0.60, *p *< 0.001), with no effect of perceptual distinctiveness or of the interaction between stimulus category and distinctiveness.

**Figure 3:**
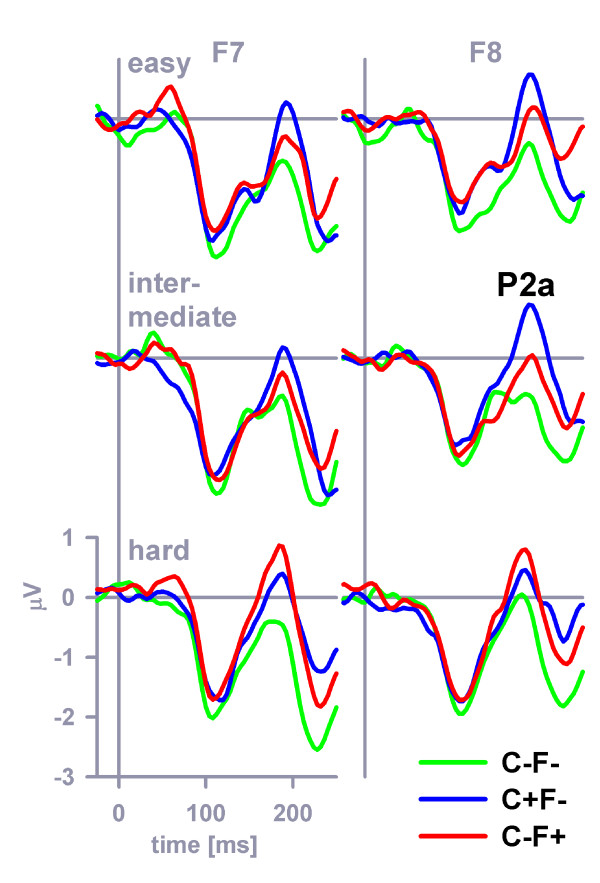
**P2a at frontolateral electrodes**. The grand-average P2a evoked by color-distractors, form-distractors and standard distractors at frontolateral electrodes (F7 = left panel, F8 = right panel).

Figure 3 plots grand-average ERPs at frontolateral electrodes (F7, F8) that were obtained in response to the three types of non-target stimuli (C+F-, C-F+, C-F-). The two types of feature-overlap distractors (+0.3 μV mean amplitude at frontolateral electrodes in the latency range 180–188 ms) generally evoked a more pronounced P2a at frontolateral electrodes than did the standard distractor (-0.6 μV mean amplitude at frontolateral electrodes in the latency range 180–188 ms), an effect that can be easily discerned by inspection of Figure 3. This observation was confirmed by a three-way stimulus category (feature-overlap distractors, standard distractors) × distinctiveness × electrodes ANOVA (stimulus category effect, *F *(1, 23) = 28.2, *MSE *= 1.89, *η*^2 ^= 0.55, *p *< 0.001). The P2a enhancement that was related to feature-overlap was modulated by perceptual distinctiveness because the color-overlap P2a was most prominent in the easy and intermediate distinctiveness conditions, whereas the form-overlap P2a was most prominent in the hard distinctiveness condition. Another three-way stimulus category (color-overlap distractor, form-overlap distractor) × distinctiveness × electrodes ANOVA confirmed the existence of this cross-over interaction (stimulus category × distinctiveness interaction effect, (*F *(2, 46) = 6.5, *MSE *= 1.05, *η*^2 ^= 0.22, *p *< 0.01, *ε *= 1.0).

### SN

**Figure 4:**
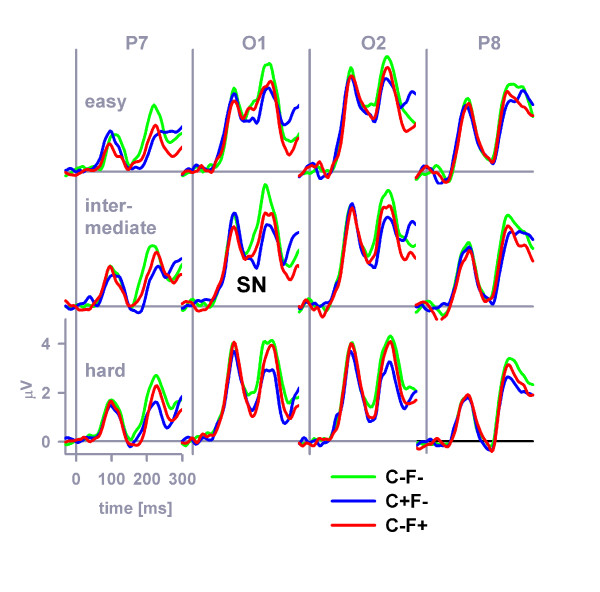
**SN at occipitoparietal electrodes**. The grand-average SN evoked by color-distractors, form-distractors and standard distractors at posterior electrodes (P7, O1, O2, P8).

Figure 4 plots grand-average ERPs at occipitoparietal electrodes that were obtained in response to the non-target stimuli, separately for the stimulus categories C+F-, C-F+, C-F-. A four-way stimulus category × distinctiveness × hemisphere (left, right) × position (O, P) ANOVA was performed on the SN mean amplitude in the latency range 152–252 ms. Stimulus category (*F *(2, 46) = 18.8, *MSE *= 2.65, *η*^2 ^= 0.45, *p *< 0.001, *ε *= 0.84), perceptual distinctiveness (*F *(2, 46) = 5.0, *MSE *= 2.85, *η*^2 ^= 0.18, *p *< 0.05, *ε *= 0.90), electrode position (*F *(1, 23) = 22.4, *MSE *= 21.3, *η*^2 ^= 0.49, *p *< 0.001) as well as the interaction distinctiveness × hemisphere (*F *(2, 46) = 3.3, *MSE *= 0.71, *η*^2 ^= 0.17, *p *< 0.05, *ε *= 0.81) exerted significant effects on the mean SN amplitude. Simple contrasts revealed that the color-overlap distractor evoked a more prominent SN compared to the standard non-target stimulus, *F *(1, 23) = 31.4, *MSE *= 2.5, *η*^2 ^= 0.58, *p *< 0.001, that the form-overlap distractor evoked a more prominent SN compared to the standard non-target stimulus, *F *(1, 23) = 24.3, *MSE *= 1.2, *η*^2 ^= 0.51, *p *< 0.001, and finally that the color-overlap distractor evoked a more prominent SN compared to the form-overlap distractor, *F *(1, 23) = 4.5, *MSE *= 2.8, *η*^2 ^= 0.16, *p *< 0.05.

**Figure 5:**
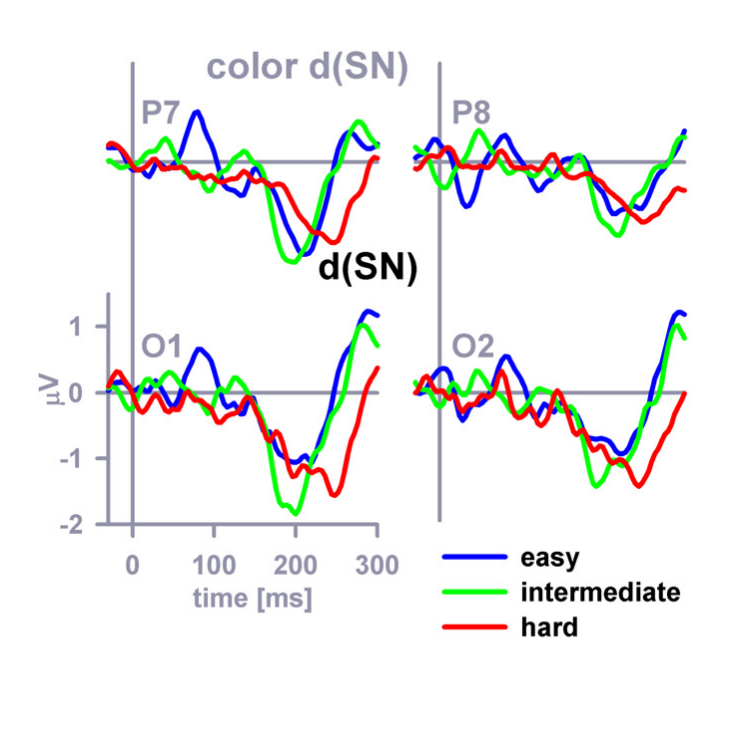
**Color-related SN difference potentials (dSNs)**. The dSN difference potentials (color-distractor – standard distractor) as they appeared before digital filtering (see Methods) at posterior electrodes (P7, O1, O2, P8).

Difference SN waves (dSNs) were computed by subtracting the standard non-target SN from the color-overlap distractor SN to further parse the color-related attentional amplification at occipitoparietal electrodes (Figure 5). Inspection of Figure 5 reveals that perceptual distinctiveness apparently affected the peak latency of the color-related dSN, but that this experimental manipulation may not have affected the peak amplitude of the color-related dSN. The observation about the peak latencies was confirmed by a three-way distinctiveness × hemisphere (left, right) × position (O, P) ANOVA that was performed on the dSN peak latencies. Perceptual distinctiveness (*F *(2, 46) = 15.3, *MSE *= 1209.5, *η*^2 ^= 0.40, *p *< 0.001, *ε *= 0.84), hemisphere (*F *(1, 23) = 12.4, *MSE *= 932, *η*^2 ^= 0.35, *p *< 0.01), electrode position (*F *(1, 23) = 14.5, *MSE *= 531, *η*^2 ^= 0.39, *p *< 0.01) proved significant (none of the interaction effects was significant). Simple contrasts revealed that the color-related dSN peaks in the perceptual easy and intermediate conditions did not differ significantly, *F *(1, 23) = 0.05, *MSE *= 628.5, *η*^2 ^< 0.01, *p *= 0.83, whereas the color-related dSN peak was delayed in the perceptual hard condition (mean = 230 ms) in comparison to the perceptual easy condition (mean = 207 ms), *F *(1, 23) = 23.5, *MSE *= 1030, *η*^2 ^= 0.51, *p *< 0.001, and to the intermediate (mean = 208 ms) condition, *F *(1, 23) = 16.2, *MSE *= 1394, *η*^2 ^= 0.41, *p *< 0.01. The observation about the peak amplitudes was confirmed by a three-way distinctiveness × hemisphere (left, right) × position (O, P) ANOVA that was performed on the color-related dSN peak amplitudes. Solely the effect of electrode position (*F *(1, 23) = 8.9, *MSE *= 0.86, *η*^2 ^= 0.28, *p *< 0.01) proved significant. All other main or interaction effects failed to reach significance.

**Figure 6:**
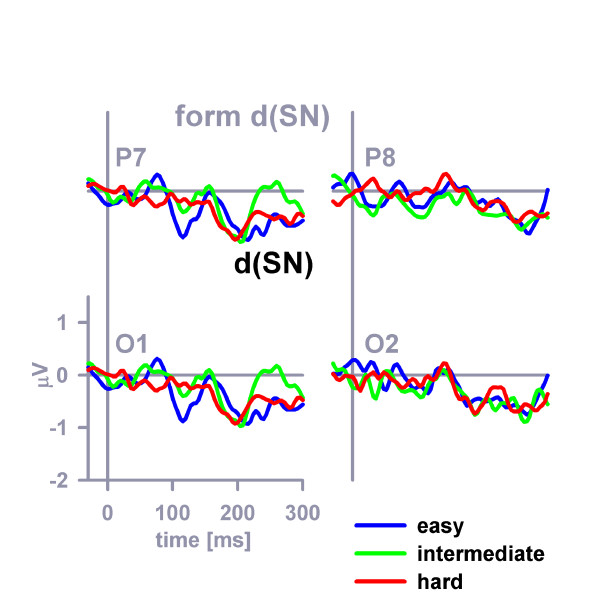
**Form-related SN difference potentials (dSNs)**. The dSN difference potentials (form-distractor – standard distractor) as they appeared before digital filtering (see Methods) at posterior electrodes (P7, O1, O2, P8).

Form-related difference SN waves (dSNs) are shown in Figure 6. Latencies and amplitudes of the form-related dSNs could not be reliably determined due to the relatively weak signal. Color-related and form-related dSN mean amplitudes (latency range 152–252 ms) were compared in a four-way stimulus category (color, form) × distinctiveness × hemisphere (left, right) × position (O, P) ANOVA. Stimulus category, *F *(1, 23) = 4.5, *MSE *= 2.8, *η*^2 ^= 0.16, *p *< 0.05 (color-related mean dSN amplitude = -0.75 μV, form-related mean dSN amplitude = -0.46 μV), and electrode position, *F *(1, 23) = 7.2, *MSE *= 0.75, *η*^2 ^= 0.24, *p *< 0.05, but no other main or interaction effect, proved significant.

### N2c and P3a

**Figure 7:**
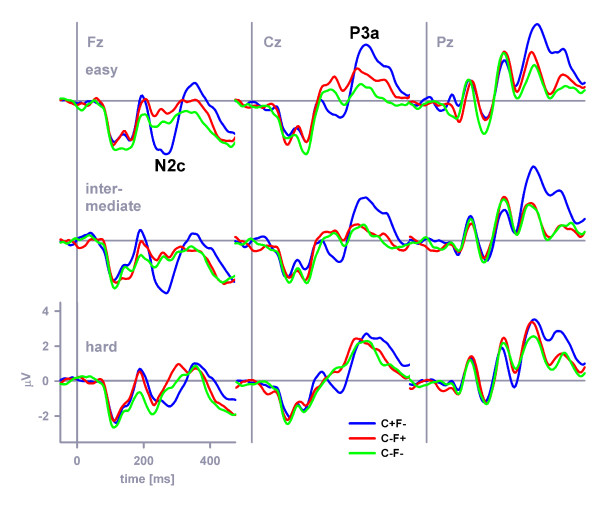
**N2c and P3a at midline electrodes**. The grand-average N2c and P3a at Fz (left panel), Cz (central panel) and Pz (right panel) evoked by the non-targets C+F-, C-F+, and C-F-. Top panel. The color-overlap distractor (C+F-) elicited a distinct N2c at Fz as well as a distinct P3a at Cz, compared with the evoked response of the other two non-targets (i.e., the form-overlap distractor (C-F+) and the standard non-target (C-F-), respectively) when colors were easily distinguishable (easy distinctiveness condition). Middle panel. Similar effects were observed when the perceptual distinctiveness of colors was at an intermediate level. Bottom panel. The color-overlap distractor (C+F-) neither elicited a distinct N2c at Fz nor a distinct P3a at Cz, compared with the evoked response of the other two non-targets (i.e., the form-overlap distractor (C-F+) and the standard non-target (C-F-), respectively) when the colors were hard to distinguish (hard distinctiveness condition).

Figure 7 plots grand-average ERPs at frontocentral midline electrodes that were obtained in response to the non-target stimuli, separately for the stimulus categories C+F-, C-F+, C-F-. A two-way stimulus category × distinctiveness ANOVA was performed on the N2c amplitude at Fz (Figure 7, left panel). Stimulus category (*F *(2, 46) = 17.0, *MSE *= 4.27, *η*^2 ^= 0.43, *p *< 0.001, *ε *= 1.0), perceptual distinctiveness (*F *(2, 46) = 6.3, *MSE *= 3.06, *η*^2 ^= 0.22, *p *< 0.01, *ε *= 0.81) as well as the interaction category × distinctiveness (*F *(4, 92) = 2.8, *MSE *= 1.72, *η*^2 ^= 0.11, *p *< 0.05, *ε *= 0.82) exerted significant effects on the N2c amplitude. Separate distinctiveness ANOVAs on the N2c amplitude at Fz were performed in each perceptual distinctiveness condition to further parse the two-way interaction. N2c amplitudes differed between the various non-target stimuli in the easy (*F *(2, 46) = 16.7, *MSE *= 2.35, *η*^2 ^= 0.42, *p *< 0.001, *ε *= 0.94) and intermediate (*F *(2, 46) = 15.2, *MSE *= 2.59, *η*^2 ^= 0.40, *p *< 0.001, *ε *= 0.92) distinctiveness conditions, whereas no amplitude difference was discernible in the hard distinctiveness condition (*F *(2, 46) = 0.9, *MSE *= 3.16, *η*^2 ^= 0.04, *p *= 0.403, *ε *= 0.82). In the easy distinctiveness condition, simple contrasts revealed that the color-overlap distractor evoked a more prominent N2c compared to the standard non-target stimulus (form-overlap distractor), *F *(1, 23) = 14.4, *MSE *= 5.54, *η*^2 ^= 0.38, *p *< 0.01, (*F *(1, 23) = 35.0, *MSE *= 3.83, *η*^2 ^= 0.60, *p *< 0.001) (Figure 7, top left panel). In the intermediate distinctiveness condition, simple contrasts revealed that the color-overlap distractor evoked a more prominent N2c compared to the standard non-target stimulus (form-overlap distractor), *F *(1, 23) = 16.7, *MSE *= 5.96, *η*^2 ^= 0.42, *p *< 0.001, (*F *(1, 23) = 33.6, *MSE *= 3.46, *η*^2 ^= 0.59, *p *< 0.001) (Figure 7, middle left panel). Finally, in the hard distinctiveness condition, simple contrasts revealed that the standard non-target stimulus (form-overlap distractor) evoked a similar N2c compared to the color-overlap distractor, *F *(1, 23) = 1.3, *MSE *= 3.86, *η*^2 ^= 0.05, *p *= 0.271, (*F *(1, 23) = 1.1, *MSE *= 7.6, *η*^2 ^= 0.05, *p *= 0.303) (Figure 7, bottom left panel).

A two-way stimulus category × distinctiveness ANOVA was performed on the P3a amplitude at Cz (Figure 7, central panel). Stimulus category (*F *(2, 46) = 14.0, *MSE *= 3.28, *η*^2 ^= 0.38, *p *< 0.001, *ε *= 0.95), perceptual distinctiveness (*F *(2, 46) = 4.4, *MSE *= 4.09, *η*^2 ^= 0.16, *p *< 0.05, *ε *= 0.96) as well as the interaction category × distinctiveness (*F *(4, 92) = 4.1, *MSE *= 2.16, *η*^2 ^= 0.15, *p *< 0.01, *ε *= 0.81) exerted significant effects on the P3a amplitude. Separate distinctiveness ANOVAs on the P3a amplitude at Cz were performed in each perceptual distinctiveness condition to further parse the two-way interaction. P3a amplitudes differed between the various non-target stimuli in the easy (*F *(2, 46) = 14.2, *MSE *= 2.6, *η*^2 ^= 0.38, *p *< 0.001, *ε *= 0.94) and intermediate (*F *(2, 46) = 10.3, *MSE *= 2.33, *η*^2 ^= 0.31, *p *< 0.001, *ε *= 0.92) distinctiveness conditions, whereas no amplitude difference was discernible in the hard distinctiveness condition (*F *(2, 46) = 0.6, *MSE *= 2.34, *η*^2 ^= 0.03, *p *= 0.513, *ε *= 0.86). Simple contrasts further revealed that the color-overlap distractor evoked a more prominent P3a compared to the standard non-target stimulus (form-overlap distractor), *F *(1, 23) = 35.9, *MSE *= 3.69, *η*^2 ^= 0.61, *p *< 0.001, (*F *(1, 23) = 10.5, *MSE *= 5.9, *η*^2 ^= 0.31, *p *< 0.01) in the easy distinctiveness condition (Figure 7, top central panel). Simple contrasts revealed that the color-overlap distractor evoked a more prominent P3a compared to the standard non-target stimulus (form-overlap distractor), *F *(1, 23) = 11.2, *MSE *= 5.16, *η*^2 ^= 0.33, *p *< 0.01, (*F *(1, 23) = 16.0, *MSE *= 4.65, *η*^2 ^= 0.41, *p *< 0.01) in the intermediate distinctiveness condition (Figure 7, middle central panel). Finally, simple contrasts revealed that the standard non-target stimulus (form-overlap distractor) evoked a similar P3a compared to the color-overlap distractor, *F *(1, 23) = 0.6, *MSE *= 5.27, *η*^2 ^= 0.03, *p *= 0.45, (*F *(1, 23) = 1.0, *MSE *= 4.29, *η*^2 ^= 0.04, *p *< 0.321) in the hard distinctiveness condition (Figure 7, bottom central panel).

## Discussion

ERPs revealed neural processes related to visual feature analysis, selective attention and conflict-related processing during cross-dimensional go-nogo choices. Firstly, target identification was accompanied by the emergence of both, P2a and P3b. The relative feature salience was reflected in P2a amplitude that was recorded at frontolateral electrodes in response to feature-overlap distractors. Secondly, target-compatible features captured attention more than target-incompatible features as revealed by dSNs. We conclude that intra-dimensional attentional capture was goal-contingent. Color-related dSNs were more prominent than form-related dSNs, suggesting that the color property was possibly more preeminent than the form property. There were no measurable changes of cross-dimensional selection priorities when color discriminability was altered. Thirdly, peak latencies of color-related dSNs provide a chronometric measure of the duration of attention-related neural processing. Fourthly, ERPs recorded over the frontocentral scalp (N2c, P3a) showed that color-overlap distractors required more late selection than form-overlap distractors. Finally, the need for additional late selection of color-overlap distractors was severely reduced when color discriminability decreased. The theoretical relevance of these results is discussed below.

It is well-known that the P3b emerges in response to target stimuli in oddball tasks (see [37] for review). The P3b and the prefrontal P2a showed similar sensitivity to target stimuli (see also [18,21,22]). It has been proposed that the P2a is related to the perceptual analysis of stimuli, possibly reflecting a cross-dimensional feature detection process [22]. Alternatively, recent papers promoted the role of top-down processing in visual object recognition (e.g. [38,39]). Target sensitivity of the P2a may contribute to top-down facilitation of object recognition. A partially analyzed version of the visual input is projected from visual areas to the prefrontal cortex [18,21,39]. This process, in turn, facilitates object recognition through intracortical feedback by limiting the number of objects that need to be considered (a mechanisms called the "initial guess", [38]).

Feature salience was reflected in the amplitude of the P2a, evoked at frontolateral electrodes by color-overlap and form-overlap distractors. In particular, when colors were easy to distinguish (i.e., in the easy and intermediate color distinctiveness conditions), color-related P2a (obtained from color-overlap distractors) was enlarged as compared to form-related P2a (obtained from form-overlap distractors). However, when color discriminability decreased (i.e., in the hard color distinctiveness condition), color-related P2a was attenuated relative to form-related P2a (cf. Figure 3). These results are compatible with the idea that P2a reflects neural processing related to an early, preliminary target identification process [18,21,38,39]. The crossover interaction between the color-related and form-related P2a amplitudes and color discriminability conditions suggests that adaptive changes of selection priorities – based on the relative salience of feature dimensions – are part of this processing.

As outlined in the introduction, the issue of reflexive versus voluntary control has traditionally been important for theories of selective attention [2-4]. A behavioral study by Folk and colleagues [10] is an important precursor of our study because distractor properties and the properties used to find the target were analyzed in both studies. When subjects in Folk et al.'s study looked for abrupt onset targets, abrupt-onset distractors, but not color distractors, captured attention. When subjects were looking for color targets, color distractors, but not abrupt-onset distractors, captured attention. Folk et al. [10] concluded that not the stimulus properties of the distractors per se, but the relationship of distractor properties and target-defining properties, determines attentional capture. Folk et al. [10] proposed that cognitive goals determine attentional control settings in advance (off-line). The appearance of stimuli matching that setting capture attention (on-line) without further involvement of other cognitive processes [3,9].

This idea is the theoretical background of analyzing SNs and dSNs in our study. The attentional control setting necessarily specified two cross-dimensional target properties, namely its color and form. The various types of distractors (i.e., C+F-, C-F+, or C-F-, respectively) differed only with regard to the relationship of distractor properties and target-defining properties. For example, color-related dSN reflects goal-contingent attentional capture induced by the presence of a target-compatible color-feature in the C+F--distractor in comparison to the presence of a target-incompatible color-feature in the C-F--distractor. Comparisons were made using identical physical stimuli under different conditions to isolate purely attentional effects (cf. Methods). The feature-related dSNs thus reflect purely goal-contingent, rather than reflexive, attentional capture by target-compatible distractor properties. Our dSN results are in general agreement with those of many earlier ERP studies [34,35,40-43] and functional magnetic resonance imaging (fMRI) studies [2,5,9] examining top-down attentional modulation.

The analysis of feature-related dSNs suggests that color was probably the prior-ranking property of the attentional control setting. No evidence for dynamic adaptation of selection priorities in accordance with the relative salience of the feature dimensions could be discerned from the dSNs. This result contrasts with frontolateral P2a findings, suggesting that occipitoparietal dSN and prefrontal P2a are dissociable components of the ERP.

The latency of the peak of the color-related dSN provides a chronometric measure of the duration of attention-related neural processing. On average, the color-related dSN peaks occurred 207 ms (easy discriminability condition), 208 ms (intermediate condition) and 230 ms (hard condition) after stimulus onset. Thus, the color-related dSN peak latency effect yields a full explanation of the effect of the manipulation of color distinctiveness on average response times which were 390 ms (easy discriminability condition), 387 (intermediate condition) and 406 ms (hard condition). We propose that the peak of the color-related dSN provides a suitable approach towards chronometrically localizing [44] the neural effects of manipulations of color discriminability. The chronometric effect (~200–230 ms) lies in-between early sensory encoding (cf. the latency of the P1 component of the ERP) and components of the ERP that are related to late selection (see below). Its cognitive correlate is most likely the goal-contingent attentional modulation of late perceptual analysis, in this case color analysis. A number of alternative chronometric measures for late perceptual analysis based on ERPs has recently been described [41,42,45,46].

Both feature-overlap distractors, but not the standard distractor, possessed the potential to provoke response conflicts, due to the coexistence of "go" and "nogo" signals (Figure 1). As outlined in the introduction, the neural mechanisms of conflict-related processing can be analyzed by comparing N2c and P3a in response to color-overlap and form-overlap distractors, respectively, with N2c and P3a in response to the standard distractor.

Hansen and Hillyard [47] distinguished three functional modes of multidimensional selection. Firstly, they proposed a *hierarchically dependent *mode, in which the selection in one dimension (e.g., form) depends on whether another dimension (e.g., color) has a target-compatible feature. In this mode, ERPs to target-compatible and target-incompatible features of one dimension would show no difference *if *the feature of the other dimension is target-incompatible. With respect to N2c and P3a, these findings were obtained in easy and intermediate color distinctiveness conditions: The amplitudes of both ERP components in response to the form-overlap distractor (i.e., C-F+) and the standard distractor (i.e., C-F-) were indistinguishable. This result suggests that stimuli have received no goal-contingent processing *if *the color feature was target-incompatible. In contrast, *if *the color feature was target-compatible, stimuli have received further goal-contingent processing, as revealed by enhanced N2c and P3a amplitudes in response to color-overlap distractors (i.e., C+F-) as compared to the corresponding amplitudes in response to the remaining distractor types.

Further, Hansen and Hillyard [47] proposed an *independent *selection mode, in which the selection of one feature does not depend on another feature being target-compatible. The independent selection mode was not supported by our data. Finally, a *holistic *mode was proposed, in which selection is based on the conjunction of relevant features. In this mode, ERPs in response to all stimuli are identical except for those possessing the relevant conjunction of features (i.e., the targets). Our N2c and P3a results suggest that this selection mode occurred in the hard color distinctiveness condition: The amplitudes of both ERP components were indistinguishable for the form-overlap distractor (i.e., C-F+), for the color-overlap distractor (i.e., C+F-), and for the standard distractor (i.e., C-F-).

**Figure 8:**
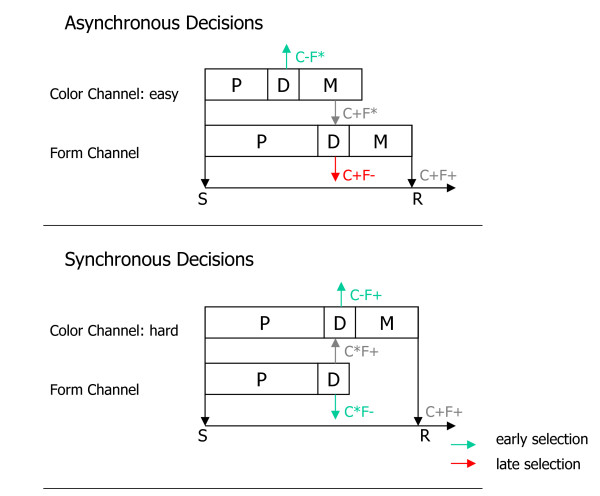
**Illustration of the decision timing model**. Top panel. Asynchronous hierarchical decisions in two separate, parallel perceptual channels (color, form) are illustrated. The color-overlap distractor, but not the other distractors, reaches response preparation in the temporal gap between the offset of the color decision and the onset of the form decision. It is therefore rejected by late selection. Bottom panel. Synchronous hierarchical decisions are illustrated. None of the distractors reaches response preparation because the color decision and the form decision overlap in time. All distractors are therefore rejected by early selection. P = duration of perceptual processes; D = duration of decisional processes; M = duration of response preparation (and execution, if adequate); S = stimulus onset; R = response; C+F+ = target stimulus; C+F- = color-overlap distractor; C-F+ = form-overlap distractor; C+F* = stimuli with target-compatible color attributes; C-F* = stimuli with target-incompatible color attributes; C*F+ = stimuli with target-compatible form attributes; C*F- = stimuli with target-incompatible form attributes. See Discussion for details.

Figure 8 summarizes our conclusions about the temporal dynamics of cross-dimensional perceptual decision-making (see [48] for a related model) when colors were easily distinguishable (top panel). Color is rapidly analyzed (see above), and all stimuli with target-incompatible colors (i.e., the C-F* stimuli) are rejected; these stimuli receive no further goal-contingent processing. Rejection is based on *early *selection. In contrast, all stimuli with target-compatible colors (i.e., the C+F* stimuli) covertly prime the response. The form choice about the C+F*-stimuli rejects the stimuli with target-incompatible forms (i.e., the C+F- stimuli). Rejection of these stimuli is based on *late *selection because these stimuli have already primed the response. The critical assumption is that form-related decisions start after completion of color-related decisions, so that a temporal gap arises between the offset of color-related decisions and the onset of form-related decisions. In other words, we assume *asynchronous *color-related and form-related decisions in those conditions in which colors were easily distinguishable.

The bottom panel of Figure 8 illustrates perceptual decision-making when colors were hard to distinguish. Color-related and form-related perceptual analyses proceed at comparable rates. There is no temporal gap between color-related and form-related decisions. As a consequence, *early *selection of all distractors is sufficient because no stimulus has been processed to response preparation. We assume *synchronous *color-related and form-related decisions in the condition in which colors were hard to distinguish.

Hansen and Hillyard's [47] model and our decision timing model are not mutually exclusive. The decision timing model adopts the assumptions underlying the hierarchical dependent mode, which may be considered as a description of asynchronously occurring decisions, whereas the holistic mode may be considered as a description of synchronous decisions. However, the decision timing model provides a particularly economical explanation of our N2c and P3a results. Qualitatively different modes of selection are not assumed. The decision timing model postulates response decisions within parallel perceptual channels, and it explains our N2c and P3a results.

The decision timing model assumes that visual objects are first segregated perceptually into dimensional modules (here color, form), processed in parallel. It also assumes that these modules are endowed with separable response-decision processes (see Figure 8, and see also [48]). According to the model, each dimensional module registers the features from that dimension, and makes response decisions based on these features. Conflicts between the decisions (for example, the color channel selects the color distractor, but the form channel rejects it, see Figure 8) are associated with the appearance of the N2c and the P3a, depending upon the temporal asynchrony between the decisions (see Figure 8). However, we do not claim that the N2c and the P3a are similarly related to conflict-related processing. The available P3a studies [23-28] clearly point into the direction that the P3a is functionally related to attention switching. Given this, it is possible that the P3a reflects a dimensional attention switch that may be required when the dimensional decisions are in conflict with each other. If so, it is important that conflict-related dimensional attention switches were purely goal-contingent [see also [27,28]], thereby challenging the attentional switching model [26] of the P3a which posits that the P3a reflects involuntary attention switches to deviant events. Finally, the available evidence about the functional significance of the N2c clearly suggests that this ERP component is related to the resolution of the response conflict itself [28-33].

## Conclusion

A comprehensive explanation of the temporal dynamics of neural processing related to perceptual decisions should be based on a decomposition of behavioral response times into its constituent parts [44]. We stripped cross-dimensional perceptual decision-making down to its bare essentials. We showed that traditional ERP measures provide valuable tools to empirically constrain the neural chronometry of perceptual decision-making. We capitalized mainly on the excellent temporal resolution of ERPs as well as on the fact that ERPs are evoked by events that do not require behavioral responses [12]. The ERP findings were integrated into a simple decision timing model of cross-dimensional perceptual decision-making which offers plenty opportunities to guide future studies.

Recent studies in monkeys – based on single and multiple unit recordings [49-51] – and in humans – based on fMRI [52-58] – have revealed possible neural networks for perceptual-decision making. Dorsolateral prefrontal and posterior parietal contributions are critically involved in selecting actions based on instantaneous stimulus information [53,57,58]. The left dorsolateral prefrontal cortex is particularly important for perceptual decision-making [55,56]. It becomes increasingly apparent that a frontoparietal network [2,59] is crucial for perceptual decision-making.

We have identified ERP measures of neural processing supporting component processes of perceptual decision-making. Distinct classes of ERP components were related to target identification (P2a, P3b), attentional selection (dSN) and conflict-related processing (N2c, P3a). It seems feasible to disentangle the functional connectivity within the frontoparietal decision-making network by combining the chronometric ERP measures with fMRI-based spatial activation data [60-62].

## Methods

### Participants

Twenty-four volunteers participated (M = 24 years; range = 18–36 years; eleven males; twenty-two right-handed). All participants were un-medicated and neurologically unimpaired. All had normal or corrected-to-normal vision and normal hearing. Participants were either students at the University of Technology at Braunschweig or they were employees of the Klinikum Braunschweig. They were compensated with course credits or with payment (15 €). The experimental protocol was approved by the local ethical committee. After a full explanation of the nature and objectives of the experiment, a written consent statement was obtained from participants.

### Task design

**Figure 9:**
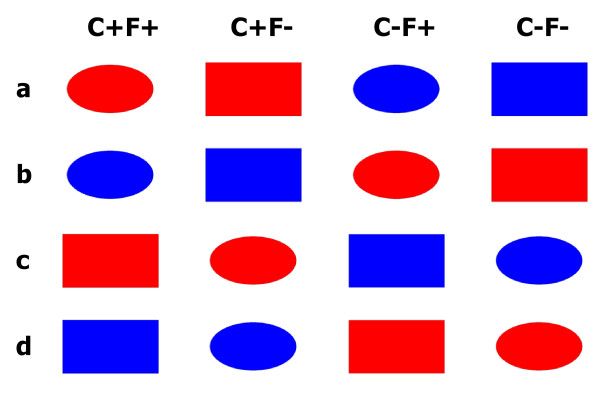
**Counterbalancing the function of the stimuli**. (**a**) When the red ellipse is the target, C+F+, the red rectangle is the color-overlap distractor, C+F-, the blue ellipse is the form-overlap distractor, C-F+, and the blue rectangle serves as standard distractor, C-F-. (**b**) When the blue ellipse is the target, C+F+, the blue rectangle is the color-overlap distractor, C+F-, the red ellipse is the form-overlap distractor, C-F+, and the red rectangle serves as standard distractor, C-F-. (**c**) When the red rectangle is the target, C+F+, the red ellipse is the color-overlap distractor, C+F-, the blue rectangle is the form-overlap distractor, C-F+, and the blue ellipse serves as standard distractor, C-F-. (**d**) When the blue rectangle is the target, C+F+, the blue ellipse is the color-overlap distractor, C+F-, the red rectangle is the form-overlap distractor, C-F+, and the red ellipse serves as standard distractor, C-F-.

Figure 9 presents the visual stimuli that were used in the target-identification ("oddball") task. Stimuli were presented one at a time in the center of a computer screen (Eizo FlexScan T766 19"; 1280 × 1024 pixels at 100 Hz presentation rate; 100 ms stimulus duration; 1150 ms inter-stimulus-interval). Stimuli were displayed on white background which extended over the complete computer screen. Stimulus presentation was controlled by the Presentation^® ^software (Neurobehavioral Systems, Albany, CA) that was installed on an IBM compatible personal computer.

The stimulus was either a red ellipse, a red rectangle, a blue ellipse, or a blue rectangle, respectively, each of which approximately subtended 2.75° × 2.25°. The four stimuli were produced by factorially combining two levels of two features. One feature was the color of the stimulus (with the two levels *blue *and *red*) and the other feature was the form of the stimulus (with the levels *ellipse *and *rectangle*).

Each participant was instructed that one stimulus was the target (denoted C+F+, i.e., the stimulus with the target-color and with the target-form) throughout the experiment. In any given trial, one out of the four stimuli was presented and participants had to decide whether or not the current stimulus equaled the target. Participants pressed the space bar on a standard computer keyboard in case that the target had been recognized (handled by the right index finger). The response had to be withheld if the stimulus was recognized as one of the non-targets. No feedback about response accuracy was provided.

There were three types of non-target stimuli, a color-overlap distractor (C+F-, i.e., the stimulus with the target-compatible color and with the target-incompatible form), a form-overlap distractor (C-F+, i.e., the stimulus with the target-incompatible color and with the target-compatible form), and a standard non-target stimulus (C-F-, i.e., the stimulus with the target-incompatible color and with the target-incompatible form). For example, if the red ellipse was the target stimulus, the red rectangle was the color-overlap distractor, the blue ellipse was the form-overlap distractor, and the blue rectangle was the standard non-target stimulus (Figure 9a). Figure 9b–d show the stimulus space as it emerged when the blue ellipse, the red rectangle, or the blue rectangle, respectively, served as target stimuli. Inspection of the four columns of Figure 9 reveals easily that each of the various stimulus types (i.e., its being the C+F+, C+F-, C-F+, or C-F-, respectively) was composed of all utilized stimuli. Adequate counterbalancing (i.e., six participants received the red rectangle as the target, another six participants received the blue rectangle as the target, etc.) thus yielded stimulus types (i.e., C+F+, C+F-, C-F+, or C-F-, respectively) that were composed of physically identical stimuli. Any comparison between averaged ERPs in response to the various stimulus types avoids physical stimulus confounds [12].

**Figure 10:**
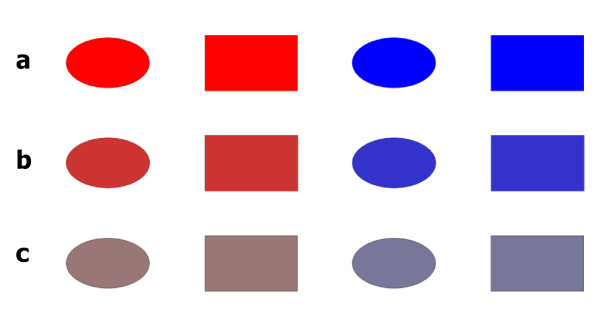
**The perceptual distinctiveness manipulation**. The perceptual distinctiveness of the colors was manipulated at three levels. (**a**) The full rich colors of red and blue stimuli (ellipse, rectangle) in the easy distinctiveness condition. (**b**) The colors of red and blue stimuli (ellipse, rectangle) in the intermediate distinctiveness condition. (**c**) The pale, nearly gray colors of red and blue stimuli (ellipse, rectangle) in the hard distinctiveness condition.

The perceptual distinctiveness of the colors was manipulated at three levels (Figure 10). This was accomplished by varying the saturation of the colors, holding their hue value (red = 0, blue = 170) and their brightness value (always 128) constant. The colors varied from rich full colors (Figure 10a) to pale, nearly gray colors (Figure 10c). The manipulation of saturation was achieved by setting the saturation value of the colors to 255 (easy condition, Figure 10a), to 145 (intermediate condition, Figure 10b), or to 35 (hard condition, Figure 10c), respectively.

Each participant performed six blocks of 192 trials each (6 × 192 = 1152 trials overall). Blocks were divided by short brakes (lasting two or three minutes). The various stimulus types (i.e., its being the C+F+, C+F-, C-F+, or C-F-, respectively) occurred equiprobable within blocks (192/4 = 48 trials). The order of succession of the stimulus types was random. The level of color distinctiveness was maintained across two consecutive blocks. Thus, each stimulus type occurred in 96 trials (2 × 48 = 96 trials) within each level of color distinctiveness. Color distinctiveness was manipulated blockwise. The order of succession of the three levels of color distinctiveness (easy, intermediate, hard) was counterbalanced across participants according to a Latin square design.

Participants were instructed that they would receive four different types of stimuli in rapid sequence, and that one of the stimuli would be the target (e.g., the "red ellipse"). They were informed about the randomness of the stimulus sequence, and they were asked to respond as fast as possible without committing errors. Participants received twenty-four practice trials in the run-up to the experiment. The target identification task that was performed on the practice stimuli was based on the number (one or two) and the spatial orientation (towards the left or towards the right) of green bars.

### Electrophysiology

Continuous EEG was recorded by means of another IBM compatible personal computer, a QuickAmps-72 amplifier (Brain Products, Gilching, Germany) and the BrainVisionRecorder^® ^(Brain Products, Gilching, Germany) software from frontal (F7, F3, Fz, F4, F8), central (T7, C3, Cz, C4, T8), parietal (P7, P3, Pz, P4, P8), occipital (O1, O2), and mastoid (M1, M2) sites. Ag-AgCl EEG electrodes were used. They were mounted on an EasyCap (EasyCap, Herrsching-Breitbrunn, Germany). Electrode impedance was kept below 10 kΩ. All EEG electrodes were referenced to average reference. Participants were informed about the problem of non-cerebral artifacts and they were encouraged to reduce them [63]. Ocular artifacts were monitored by means of bipolar pairs of electrodes positioned at the sub- and supraorbital ridges (vertical electrooculogram, vEOG) and at the external ocular canthi (horizontal electrooculogram, hEOG). The EEG and EOG channels were amplified with a bandpass of 0.01 to 30 Hz and digitized at 250 Hz.

Offline analysis was performed by means of the BrainVisionAnalyzer^® ^software (Brain Products, Gilching, Germany). Semi-automated artifact rejection was performed before averaging to discard trials during which an eye movement or any other non-cerebral artifact occurred (maximum allowed voltage step per sampling point: 50 μV; maximum allowed amplitude difference: 200 μV; minimum allowed amplitude: -200 μV; maximum allowed amplitude: 200 μV; lowest allowed activity (max-min, interval length 100 ms): 0.5μV). Ocular correction included semi-automatic blink detection and the application of an established method for ocular artifact removal [64]. The EEG was then divided into epochs of 1000 ms duration, starting 100 ms before the onset of the stimuli. The pre-stimulus baseline of 100 ms was subtracted from the sampling points. Deflections in the averaged EOG waveforms were small, which indicated good maintenance of fixation. The averaged EEG waveforms were re-referenced to the average of the left and right mastoids. No digital filtering was applied to the data.

### Data analysis

Task performance was quantified in two ways: Firstly, the median of the response speed at each level of color distinctiveness was computed for each individual participant, and these median individual response times (RTs) were subjected to statistical analysis. Secondly, the accuracy of the behavioral responses was computed at each level of color distinctiveness for each individual participant. The percentage of hits was computed for the target stimuli (C+F+). Percentages of correct rejections were separately computed for each non-target stimulus type (C+F-, C-F+, or C-F-, respectively). Finally, the percentage of correct rejections was computed as an average across all three non-target stimulus types. All these various percentages were transferred into the arsin transformation prior to statistical analysis.

ERPs were averaged separately for all combinations of perceptual distinctiveness and stimulus type (target, C+F+, non-target, i.e. C+F-, C-F+, C-F-). In addition, the non-target ERPs were collapsed across the three non-target stimulus types. Mean amplitudes of the P3b in response to the targets and to the non-targets were measured in the latency window 376 ms (peak latency) ± 4 ms with respect to the pre-stimulus baseline period at electrode Pz at which the P3b is usually maximal. Mean amplitudes of the P2a in response to the targets and to the non-targets were measured in the latency window 192 ms (peak latency) ± 4 ms with respect to the pre-stimulus baseline period at electrode Fz. Mean amplitudes of the P2a evoked by each of the non-target stimuli (i.e. by C+F-, C-F+, C-F-) were measured in the latency window 184 ms (peak latency) ± 4 ms with respect to the pre-stimulus baseline period at frontolateral electrodes (F7, F8).

Mean amplitudes of the SN evoked by each of the non-target stimuli (i.e. by C+F-, C-F+, C-F-) were measured in the latency window 152–252 ms with respect to the pre-stimulus baseline period at occipitoparietal electrodes (O1, O2, P7, P8). Color-related and form-related difference SN waveforms (dSNs) were computed by subtracting the standard waveform from the color-overlap and the form-overlap waveform, respectively, separately for each participant within each of the three perceptual distinctiveness conditions. Following digital low-pass Butterworth zero phase filtering (8 Hz cutoff, 48 db/oct) of the color-related dSN, the latencies and amplitudes of the peak of the color-related dSNs were picked within a latency window between 152 and 300 ms.

Mean amplitudes of the N2c evoked by each of the non-target stimuli (i.e. by C+F-, C-F+, C-F-) were measured in the latency window 268 ms (peak latency) ± 4 ms with respect to the pre-stimulus baseline period at electrode Fz at which the N2c was maximal. Mean amplitudes of the P3a evoked by each of the non-target stimuli (i.e. by C+F-, C-F+, C-F-) were measured in the latency window 340 ms (peak latency) ± 4 ms with respect to the pre-stimulus baseline period at electrode Cz at which the P3a was maximal.

Performance measures and the ERP amplitude measures were subjected to repeated measures analyses of variance (ANOVAs) using the Greenhouse-Geisser correction. The results of the univariate tests are provided, using a format which gives the uncorrected degrees of freedom, the corrected *p *value, and *ε *[63]. The mean square error, *MSE*, and, *η*^2^, a measure of effect size, are provided. The various performance measures were subjected to separate one-way ANOVAs with perceptual distinctiveness of the color dimension (easy, intermediate, hard) serving as within-subject factor. The mean amplitudes of the considered ERP components (P3b, P2a, SN, N2c, P3a) were subjected to separate ANOVAs, with stimulus category (e.g., target, collapsed non-target etc.), perceptual distinctiveness, hemisphere (left, right, where appropriate) and electrode position (where appropriate) serving as within-subject factors. In addition, the peak latencies and amplitudes of the color-related dSN were subjected to two separate ANOVAs. A significance level of *α *= .05 was predetermined.

## Authors' contributions

BK conceived and designed the study, programmed the experiment, collected the data, accomplished most of the data analyses and drafted the manuscript. ST accomplished some of the data analyses and contributed to the manuscript. CM and KW contributed to the manuscript. All authors read and approved the final manuscript.
